# Characterization of vesicle-associated exported immunodominant antigens of the human pathogen *Babesia duncani*

**DOI:** 10.1128/iai.00763-25

**Published:** 2026-06-03

**Authors:** Pallavi Singh, Anasuya C. Pal, Jae-Yeon Choi, Meenal Chand, Pratap Vydyam, Geeta Kumari, Choukri Ben Mamoun

**Affiliations:** 1 Section of Infectious Diseases, Department of Internal Medicine, Yale School of Medicine 12228 , New Haven, Connecticut, USA; 2 Department of Microbial Pathogenesis, Yale School of Medicine 12228 , New Haven, Connecticut, USA; 3 Department of Pathology, Yale School of Medicine 12228 , New Haven, Connecticut, USA; University of California Davis, Davis, California, USA

**Keywords:** human babesiosis, *Babesia*, parasite, Apicomplexa, vesicles, *B. duncani-*derived vesicles (BDVs)

## Abstract

Human babesiosis is an emerging disease caused by intraerythrocytic *Babesia* parasites, primarily transmitted through tick bites. Among the species of *Babesia* that infect humans, *Babesia duncani* causes severe disease in murine models, associated with excessive cytokine responses that contribute to immunopathology. The parasite factors that mediate host immune activation remain poorly defined. Here, we show that *B. duncani*, similar to *B. microti*, exports antigens through vesicle-mediated pathways. Screening a panel of 105 known and predicted secreted proteins using sera from *B. duncani*-infected and uninfected mice identified multiple immunodominant antigens. We further characterized two representative antigens and demonstrate using differential centrifugation, vesicle enrichment, immunofluorescence microscopy, and transmission electron microscopy that these antigens localize to vesicles released by *B. duncani*-infected erythrocytes into the extracellular environment. These findings reveal vesicle-mediated antigen export as a key feature of *B. duncani* infection and suggest a pathway through which parasite-derived antigens may be delivered to the host immune system. Such findings may guide future studies aimed at developing diagnostic or vaccine strategies.

## INTRODUCTION

Tick-borne diseases (TBDs) are an increasing public health concern in the United States, with over 490,000 cases annually, now surpassing mosquito-borne infections (1). This rise is driven by climate change, expanding wildlife hosts, and especially deforestation, which alters ecosystems and facilitates tick spread. The rise in TBDs highlights an urgent need for improved surveillance, diagnostics, and preventive measures as these diseases are often underdiagnosed and can cause severe illness in humans.

Among TBDs, human babesiosis is a growing threat caused by *Babesia* spp., intraerythrocytic protozoan parasites of the phylum Apicomplexa. Unlike other apicomplexans, such as *Plasmodium*, *Toxoplasma*, or *Cryptosporidium*, *Babesia* is tick-transmitted, zoonotic, and is increasingly prevalent in North America. Several *Babesia* species can infect humans, with varying geographic distributions and virulence. In the United States, *Babesia microti* is the most common agent (2), while in Europe, *Babesia divergens* is a major cause of bovine babesiosis and occasional fulminant human infection (typically in asplenic patients) (3, 4). All human-infective *Babesia* can cause illness ranging from asymptomatic to severe or even fatal disease, especially in the elderly, splenectomized, or immunocompromised individuals (5). Babesiosis, thus, represents an increasingly significant parasitic disease in regions where tick vectors are prevalent. There is no vaccine available to prevent human babesiosis caused by any *Babesia* species. Prevention relies on tick control and personal protection, which are not always effective. Species-specific diagnostic tools that distinguish active from past infections have only recently been developed (6–10).

While *B. microti* is responsible for most human babesiosis cases, *B. duncani* has emerged as a potentially highly virulent species. Identified in western North America, *B. duncani* is linked to severe and fatal infections, especially in immunocompromised or elderly patients (11–13). In experimental models, *B. duncani* infection results in uniformly lethal outcomes in C3H/HeJ mice even at very low inocula, indicating extreme susceptibility due to an uncontrolled proinflammatory response (cytokine storm) rather than parasite burden (14, 15). In contrast, C57BL/6 mice exhibit resistance to low-dose infections by effectively controlling parasite replication, but they ultimately succumb to high-dose inoculations when parasite burdens become overwhelming (14, 16). These host-specific differences indicate that *B. duncani* is capable of causing severe disease through both immunopathology in highly susceptible hosts and high parasitemia in otherwise immunocompetent hosts. Currently, our knowledge about the parasite’s development in erythrocytes, antigenic landscape, immune interactions, and pathogenesis remains limited. Identifying parasite antigens that elicit robust immune responses is critical for understanding host-pathogen interactions in *B. duncani* infection, enabling discovery of effective immune targets, vaccine design, and improved immunodiagnostic tests. To date, few *B. duncani* antigens have been characterized (6), and it is unclear which parasite proteins trigger a strong humoral immune response during infection (17).

In this study, we demonstrate vesicle-mediated protein export in *B. duncani*. We screened a library of known and predicted secreted proteins using sera from susceptible C3H/HeJ mice and resistant C57BL/6 mice, identifying 11 immunodominant proteins that consistently triggered strong IgG responses. Subcellular localization studies on two selected antigens revealed that they are associated with extracellular vesicles released into host red blood cells (RBCs). The secretion method of the remaining antigens could not be tested due to lack of specific antisera, but they remain attractive candidates for future studies.

## MATERIALS AND METHODS

### *In vitro* culture of *B. duncani* in human erythrocytes

*B. duncani* WA-1 clone 301 parasites were maintained in A^+^ human erythrocytes (American Red Cross) using DMEM/F12 (Dulbecco’s modified Eagle medium/F12 Nutrient Mix; 11320033) supplemented with 20% fetal bovine serum (Gibco; A5256801).

### Infection of C3H/HeJ and C57BL/6 mice with *B. duncani* WA-1

Female C3H/HeJ and C57BL/6J mice (5–6 weeks old, Jackson Laboratory) were acclimatized for 1 week before infection. Mice were infected via retro-orbital intravenous injection with 1 × 10⁴ *B. duncani* WA-1 strain using blood collected from a previously infected C3H/HeJ mouse as the infectious inoculum. Post-infection, blood samples were collected by tail prick or retro-orbital bleed from day 3 onward. Giemsa-stained blood smears were examined by light microscopy at 100× magnification, counting 5,000–10,000 RBCs per smear to assess parasitemia. Mice exhibiting signs of distress were euthanized according to protocols approved by the Yale University Institutional Animal Care and Use Committee (IACUC).

### Expression of recombinant secretory *B. duncani* proteins *in E. coli*

Secreted antigens identified by Nanotrap-based proteomics (*n* = 35) and those predicted as secreted or surface-exposed (*n* = 70) were codon-optimized for expression in *E. coli*. These genes were cloned into vectors for His-, MBP-, or GST-tagged fusion protein production using *E. coli* BL21 or Rosetta DE3 strains. Expression was induced with 0.5 mM isopropyl β-D-1-thiogalactopyranoside (IPTG) for MBP- or GST-tagged and 1 mM IPTG for His-tagged constructs. In total, 78 His-, 91 MBP-, and 50 GST-tagged recombinant proteins were expressed, generating a comprehensive secretome library for downstream immunoreactivity screening with sera from uninfected and *B. duncani*-infected mice.

### Purification and preparation of *B. duncani* recombinant antigens

Glycerol stocks of *E. coli-*expressing *B. duncani* antigens in pET21 (His-tagged), pGEX (GST-tagged), or pMAL (MBP-tagged) vectors were used to inoculate primary cultures (3 mL Luria broth [LB] medium). These primary bacterial cultures were grown to saturation (overnight [16 h] at 37°C) and diluted 1:3 into secondary cultures (1 mL/well) in Nunc 96 DeepWell Polystyrene Plates (Catalog number 278606), either uninduced or induced with 1 mM IPTG. Once the OD_600_ of the secondary cultures reached 0.6, the His-tagged proteins were induced for 3 h at 37°C; GST- and MBP-tagged proteins were induced overnight (16 h) at 16°C. Plates were centrifuged (2,000 × g, 20 min, 4°C), supernatants were discarded, and pellets were resuspended in 1 mL lysis buffer, followed by gentle mixing and lysis for 1 h at room temperature.

Lysis buffer composition for *E. coli-*expressing MBP-tagged and GST-tagged proteins : 50 mM Tris-HCl (10812846001, Sigma) pH 8.0, 150 mM NaCl (S9888-25G, Sigma), 1 mM EDTA (20-158, Sigma), 0.1% Triton X-100 (X-100, Sigma), 1 mg/mL lysozyme (L4919-500MG, Sigma), and 1× protease inhibitor (11836170001, Sigma). Lysis buffer composition for *E. coli-*expressing His-tagged proteins: 50 mM monosodium phosphate (NaH_2_PO_4_) (7558-80-7, Sigma), 300 mM NaCl (S9888-25G, Sigma), 0.1% Triton X-100 (X-100, Sigma), 1 mg/mL lysozyme (L4919-500MG, Sigma), and 1× protease inhibitor (11836170001, Sigma).

### Enzyme-linked immunosorbent assay (ELISA) to detect immunodominant *B. duncani* antigens

The lysed *E. coli* plates (obtained after the procedure above) were centrifuged (2,000 × g for 2 h at 4°C), and cleared *E. coli* lysates (100 μL/well, containing soluble tagged antigens) were used to coat high-binding Nunc MaxiSorp ELISA plates (423501, BioLegend) in triplicate. Plates were incubated overnight at 4°C and then washed three times with phosphate-buffered saline containing 0.05% Tween-20 (PBS-T) (P2287, Sigma). The plates were blocked with 200 μL/well with 5% bovine serum albumin (BSA) in PBS for 1.5 h at 37°C, then washed four times with PBS-T, and incubated with preheated (56°C, 30 min) mouse serum (either from *B. duncani*-infected C3H/HeJ or C57BL/6 mice) diluted 1:1,000 in 1% BSA-PBS (100 μL/well) for 2 h at room temperature. After four washes with PBS-T, HRP-conjugated anti-mouse IgG (1:5,000 in 1% BSA-PBS) was added (100 μL/well) for 1 h at room temperature. Plates were washed four times and then developed with 50 μL/well TMB (3,3',5,5′-tetramethylbenzidine) substrate for 10 min. The reaction was stopped by addition of 50 μL/well of 0.1 N HCl, and the optical density at 450 nm was measured using a BioTek Synergy Mx plate reader. ELISA cutoffs were defined using preimmune mouse sera as the mean OD_450_ values plus three standard deviations. Samples with readings above this threshold, corresponding to OD_450_ > 0.2, were considered positive and specific.

### Antibody generation and purification

Polyclonal rabbit antibodies against 13 secreted antigens were raised (Cocalico Biologicals, Inc., Pennsylvania). The sera were used for polyclonal antibody purification via Protein G column chromatography (Cytiva; 17040401) using the manufacturer’s protocol. Briefly, sera were applied to pre-equilibrated Protein G columns, washed to remove nonspecific proteins, and bound antibodies were eluted and neutralized. Fractions were pooled, and antibody concentrations were quantified using a NanoDrop spectrophotometer (Agilent, BioTek Synergy H1, TAKE3-SN).

### Isolation of host RBC-derived and parasite-derived vesicle fractions

To isolate extracellular vesicle fractions, *B. duncani in vitro* culture supernatants and *B. duncani*-infected RBC lysates were processed by a series of centrifugation and ultracentrifugation steps. Briefly, uninfected or *B. duncani*-infected human RBCs (15% parasitemia) were centrifuged at 500 × *g* for 30 min to remove intact cells. For extracellular vesicle isolation from the culture supernatant, the cleared supernatants (S) were ultracentrifuged at 120,000 × *g* for 14 h at 4°C using a Sorvall MTX 150 micro-ultracentrifuge equipped with an S52-ST swinging bucket rotor (Thermo Fisher Scientific). After ultracentrifugation, the pellet (Up) containing vesicle-enriched material and the corresponding supernatant (Us) were carefully collected and used for immunoblotting.

To obtain hemolysate fractions containing *B. duncani*-derived or uninfected RBC-derived vesicles, *B. duncani in vitro* culture pellets (15% parasitemia) and uninfected RBC pellets were lysed with 0.05% saponin in phosphate-buffered saline (PBS) for 10 min on ice, followed by centrifugation at 500 × *g* for 10 min to remove the cell debris. The lysates were then subjected to ultracentrifugation under the same conditions described above (120,000 × *g* for 14 h at 4°C). The resulting pellet (Hp) and supernatant (Hs) were subsequently separated and used for immunoblotting.

### Immunofluorescence assays

Thin blood smears (10%–15% parasitemia) from *B. duncani in vitro* cultures were fixed with pre-chilled methanol for 10 min at -20°C, air-dried, and blocked with 3% BSA in PBS for 1 h at room temperature. For the first set of IFAs, the slides were incubated for 1 h at room temperature with primary rabbit polyclonal antibodies (anti-BdV235, anti-BdV234) (1:500) and mouse monoclonal anti-band3 antibody (Sigma; B9277-100UL) (1:500), washed with PBST and PBS, and incubated for 1 h with Alexa Fluor 488-conjugated goat anti-rabbit IgG (Thermo Fisher Scientific, A-11008; 1:500 dilution) and Alexa Fluor 594-conjugated anti-mouse IgG (Thermo Fisher Scientific, A-11005; 1:500 dilution). For the second set of IFAs, the slides were incubated for 1 h at room temperature with anti-BdV19 mouse serum (1:500), washed with PBST and PBS, and incubated for 1 h with Alexa Fluor 488-conjugated goat anti-mouse IgG (Thermo Fisher Scientific A-11001; 1:500 dilution). After washing, coverslips were mounted with VECTASHIELD mounting medium containing DAPI (Vector Laboratories, H-1200-10). Slides were examined on a Nikon ECLIPSE TE2000-E fluorescence microscope (100 X oil immersion objective). Fluorescence signals for Alexa Fluor 488, Alexa-Fluor 594, and DAPI were detected at respective excitation wavelengths. Images were captured (1,392 × 1,040 pixels; MetaVue software) and analyzed with ImageJ.

### Immunodetection of secreted antigens in *B. duncani*

Supernatant and pellet fractions from serial differential centrifugation (2,000 × *g* for 10 min, 10,000 × *g* for 40 min, 100,000 × *g* for 1.5 hours, and 150,000 × *g* for 14 hours) as well as Us, Up, Hs, and Hp fractions (supernatant and pellet fractions from uninfected human RBCs and *B. duncani*) were mixed with Laemmli sample buffer (BIO-RAD, 1610747), denatured at 95°C for 5 min, and resolved using 4%–12% gradient SDS-PAGE. Proteins were transferred to nitrocellulose membranes and blocked in 5% non-fat dry milk dissolved in PBS for 1 h at room temperature. Membranes were incubated overnight at 4°C with either rabbit polyclonal antibodies (anti-BdV234, and anti-BdV235; dilution 1:250), or anti-BdV19 mouse serum (dilution 1:250), washed in PBST, and incubated for 1 h at room temperature with either HRP-conjugated goat anti-rabbit IgG (Cell Signaling, 7074; 1:5,000 dilution) or goat anti-mouse IgG (Cell Signaling, 7076; 1:5,000 dilution). Blots were washed and developed using the SuperSignal West Pico PLUS chemiluminescent substrate (Thermo Scientific, 34577). Images were captured using a LI-COR Odyssey-Fc imaging system.

### Alternative method for isolation of extracellular vesicles from *B. duncani in vitro* culture supernatants

*B. duncani* cultures initiated at 1% parasitemia in A^+^ human RBCs (5% HC) were grown for 5 days to reach 10%–15% parasitemia. Extracellular vesicles (EVs), including exosomes, were isolated from supernatants using the ExoQuick ULTRA EV Isolation Kit (System Biosciences, EQULTRA-20A-1). Supernatants, clarified by centrifugation (3,000 × *g*, 15 min), were mixed with ExoQuick reagent and incubated at 4°C overnight. EVs were pelleted by centrifugation, resuspended in Buffer B, and further purified using column resin by centrifugation at 1,000 × *g*. This two-step protocol yielded high-purity EV samples suitable for downstream analyses.

### High-pressure freezing and freeze-substitution Epon section and labeling

*B. duncani*-infected mouse RBCs (15% parasitemia) were fixed in 4% paraformaldehyde (PFA) and then subjected to high-pressure freezing at 2,000 psi (Leica HMP100). Freeze substitution (Leica AFS) was performed at −95°C in 1% osmium tetroxide, 1% glutaraldehyde, and 1% water in acetone for 10 h. The temperature was gradually increased to −20°C over 12 h and then to 4°C for 2 h. After washing in acetone, samples were embedded in Durcupan resin and cured at 60°C for 24 h. Ultrathin sections (~60 nm) were obtained with a Leica UltraCut UC7 and mounted on nickel grids with formvar and carbon film.

### Cryo-immuno-EM embedding and antibody labeling

*B. duncani*-infected human RBCs were fixed in 4% paraformaldehyde (PFA) and 0.125% glutaraldehyde (room temperature, 15–30 min), then in 4% PFA (4°C, 1 h), washed, embedded in 10% gelatin, and processed in 2.3 M sucrose overnight at 4°C. Gelatin blocks were trimmed, mounted on aluminum pins, rapidly frozen in liquid nitrogen, and sectioned (60 nm) by Tokuyasu ultracryotomy (Leica Cryo-EMUC6). Sections were mounted on carbon-/formvar-coated grids, quenched, and blocked in PBS with 0.5% BSA and 1% fish skin gelatin. For immuno-EM, grids were incubated with primary rabbit anti-BdV235 (1:150), followed by 10 nm Protein A gold secondary labeling. Grids were fixed, stained with uranyl acetate/methylcellulose, and air-dried. For negative stain and vesicle immunolabeling, samples were applied to grids, incubated with primary antibody in PBS with 1% fish skin gelatin, labeled with 5 nm Protein A gold, fixed, and stained. Imaging was done using an FEI Tecnai Biotwin transmission electron microscope (80 kV) with AMT NanoSprint15 MK2 sCMOS camera.

BdV235 immunogold labeling was quantified in images of uninfected and *Babesia duncani*-infected human erythrocytes. The micrometer-to-pixel ratio was determined in ImageJ using the image scale, and the erythrocyte boundary was manually traced to calculate the cell area. Gold particles in the erythrocyte cytosol were counted using the multi-point tool in ImageJ, excluding particles within the parasite or on the parasite plasma membrane in *B. duncani*-infected cells. Particle density was calculated as the number of gold particles divided by the cell area and shown as particles/µm^2^.

## RESULTS

### Parasite-derived vesicle formation during *B. duncani* intraerythrocytic asexual development

**Fig 1:**
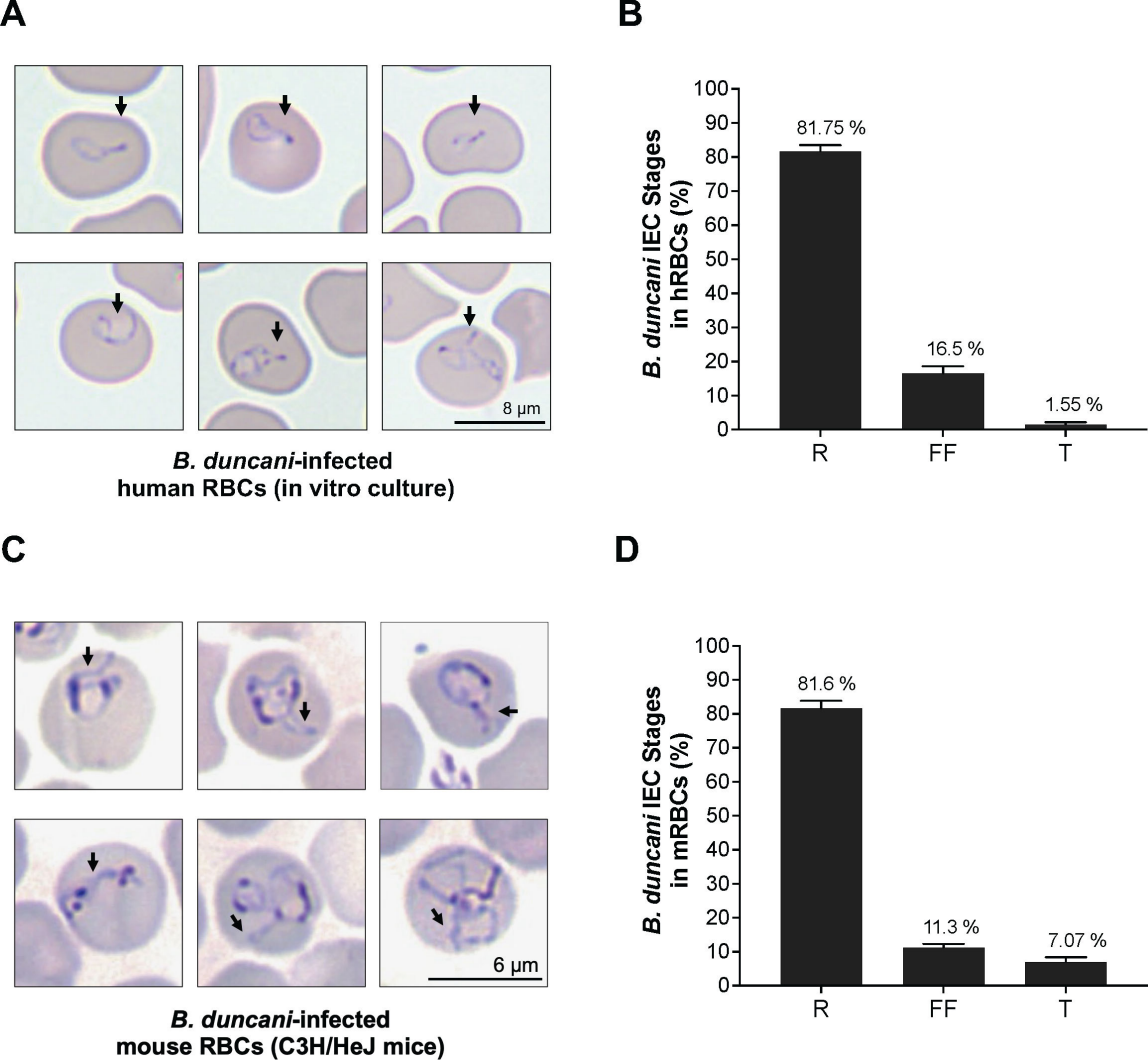
Comparative analysis of filamentous stages in *Babesia duncani*-infected erythrocytes. (**A**) Giemsa-stained images of *B. duncani* filamentous forms in human RBCs (*in vitro*). (**B**) Quantification of asexual stage rings (R), filamentous forms (FF), and tetrads (T) in human RBCs. (**C**) Representative filamentous forms in mouse RBCs (C3H/HeJ). (**D**) Quantification of stages in mouse RBCs. Data: three independent experiments, each conducted in triplicate.

To examine the morphological features associated with *B. duncani* intraerythrocytic development, we analyzed asexual parasites propagated *in vitro* in human erythrocytes and *in vivo* in mouse erythrocytes. Similar to *B. microti* (2), *B. duncani* replicates via binary fission, leading to the formation of tetrads. The majority of ring forms resemble those observed in *B. microti* and *Plasmodium* species. However, during routine propagation *in vitro* or following infection of mice, a subset of ring-stage parasites exhibited elongated filamentous extensions projecting into the host erythrocyte cytoplasm (Fig. 1A through D). These forms appeared to originate from the parasite and extend into the host cell cytoplasm.

**Fig 2:**
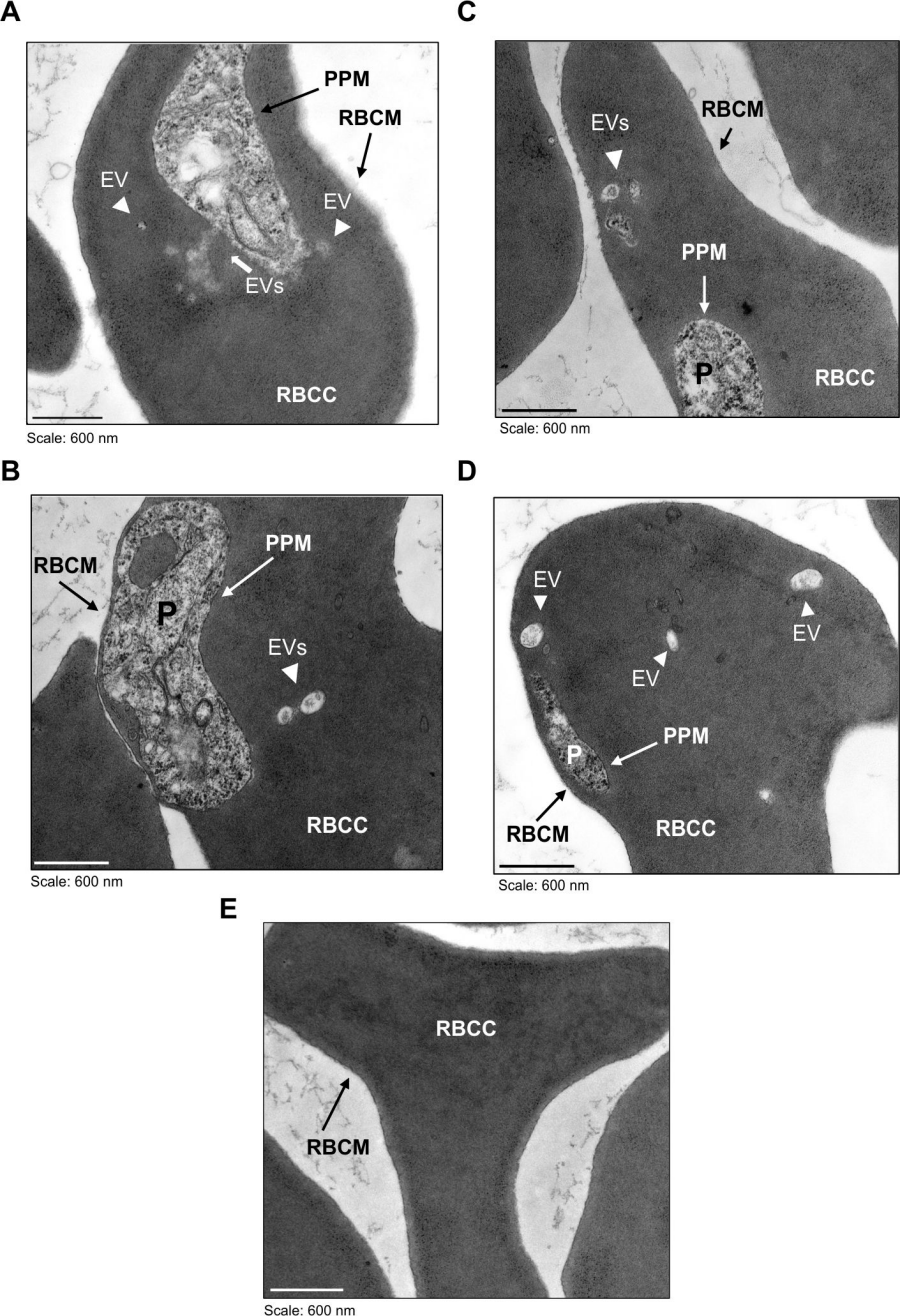
*B. duncani* generates and secretes extracellular vesicles in infected erythrocytes. (**A–D**) Ultrathin Epon sections of *B. duncani*-infected human erythrocytes showing parasite-derived extracellular vesicles (EVs). (**E**) Ultrathin Epon section of uninfected human erythrocytes. P, parasite; PPM, parasite plasma membrane; RBCC, red blood cell cytoplasm; RBCM, red blood cell membrane; EV, extracellular vesicle.

To assess whether the occurrence of these filamentous stages differed between growth conditions, we quantified their prevalence alongside rings and tetrad stages in infected human erythrocytes (~10% parasitemia) and mouse erythrocytes (~9% parasitemia) (Fig. 1B and D). Filamentous forms accounted for approximately 16% of *B. duncani* parasites in cultured human erythrocytes and ~11% of parasites in mouse erythrocytes (Fig. 1B and D). These findings indicate that their formation is intrinsic to the parasite’s development program and not an artifact of culture or the host environment. To further characterize the ultrastructural features associated with these filamentous extension structures, *B. duncani*-infected erythrocytes isolated from C3H/HeJ mice were processed by Epon embedding and transmission electron microscopy. This analysis revealed that filamentous extensions were frequently observed in close association with vesicle-like structures within the erythrocyte cytoplasm (Fig. 2A). These electron-dense vesicles contained material resembling the parasite cytoplasm, consistent with a parasite-derived origin (Fig. 2B through D). Similar vesicle-associated export structures have been reported during infection with *B. microti*, whereas extracellular vesicles released from *B. divergens*-infected erythrocytes have also been characterized (18–20).

### Identification of immunodominant antigens of *B. duncani*

**Fig 3:**
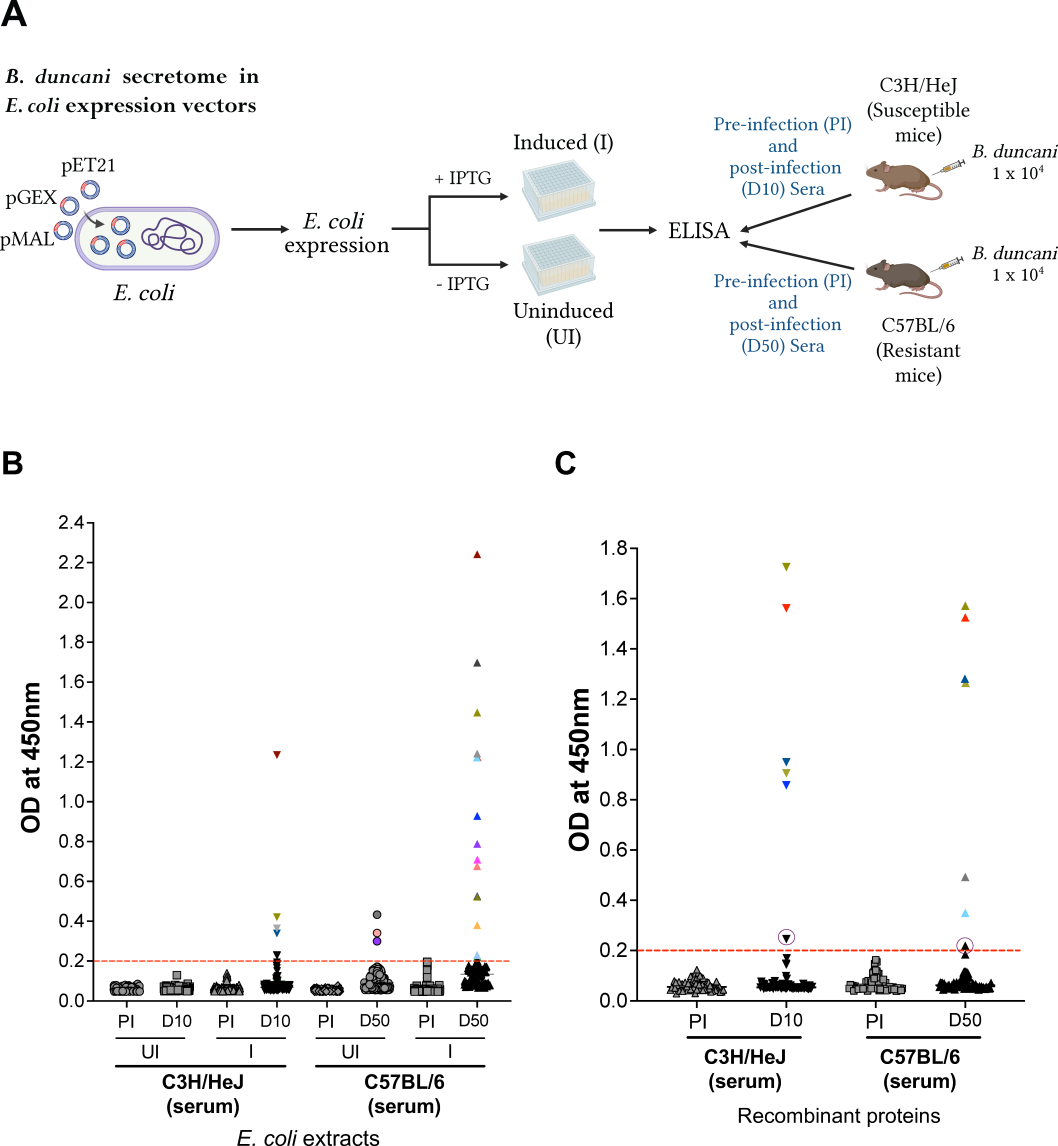
Immunodominant antigens of *B. duncani*. (**A**) Schematic of the ELISA workflow using *E. coli* lysates, induced or uninduced for His-, MBP-, or GST-tagged antigens. (**B and C**) ELISA detection (OD_450_nm) of immunodominant antigens from *E. coli* lysates (**B**) or recombinant proteins (**C**) using sera from *B. duncani*-infected C57BL/6 (D50) and C3H/HeJ mice (D10). Pre-infection serum was used as control. UI, uninduced; I, induced. Empty circle in panel **B** marks a candidate identified uniquely in this assay.

To identify antigens involved in host immune recognition, we carried out a systematic screen of known secreted (6) and predicted surface-exposed proteins from the *B. duncani* proteome (21). Predictive algorithms including SignalP, TMHMM, and PredGPI were used to screen for proteins containing N-terminal signal peptides, transmembrane domains, or GPI anchors, which are characteristic features of membrane-associated or secreted proteins. Proteins predicted to localize to intracellular organelles, as indicated by the presence of a mitochondrial targeting signal using TargetP 2.0; those smaller than 10 kDa; or those with multiple transmembrane domains lacking a continuous N-terminal extracellular region were excluded through manual curation. For proteins with only a signal peptide, the ectodomain was defined as the sequence immediately following the signal peptide. For proteins predicted to be anchored to the plasma membrane by a transmembrane domain or GPI anchor, the ectodomain was defined as the region between the end of the signal peptide and the start of the anchoring motif. Additionally, secreted proteins previously identified in a Nanotrap-based proteomic analysis of *B. duncani* were incorporated into the final list (6). The DNA regions corresponding to the ectodomains of a total of 105 proteins were codon-optimized for efficient bacterial expression and cloned into *E. coli* expression vectors to produce fusion proteins with His, MBP, or GST following induction with IPTG. Extracts from IPTG-induced and uninduced cultures were coated onto ELISA plates and probed with sera from susceptible (C3H/HeJ) and resistant (C57BL/6) mice infected with *B. duncani*. Sera collected from both of these mouse strains prior to infection (Pre-infection sera) were used as controls. Immunoreactivity was detected using HRP-conjugated goat anti-mouse secondary antibody, and the absorbance was measured at 450 nm (OD_450_) (Fig. 3A). Among the 105 candidates, 10 secreted antigens (BdWA1_002112, BdWA1_002869, BdWA1_003039, BdWA1_002223, BdWA1_001369, BdWA1_002154, BdWA1_003373, BdWA1_002224, BdWA1_000266, and BdWA1_000750) showed strong reactivity with sera from both mouse strains using extracts from IPTG-induced cultures but not uninduced cultures (Fig. 3A and B, Table 1), confirming their immunodominance. Similar to our analysis using bacterial total cell extracts, recombinant proteins were also purified and used as antigens in ELISA assays with sera from susceptible (C3H/HeJ) and resistant (C57BL/6) mice infected with *B. duncani*. Of the 105 proteins expressed in *E. coli*, only 51 proteins could be purified in sufficient amounts for ELISA assays. When using purified recombinant proteins as the antigen source in ELISA, five immunodominant antigens were detected using the sera of C3H and C57BL/6 mice, four (BdWA1_002223, BdWA1_000266, BdWA1_003373, and BdWA1_000750) of which were previously identified as immunodominant in the ELISA using the *E. coli* extract (Fig. 3B). Additionally, one immunodominant antigen (BdWA1_002873; BdV35) was identified specifically using recombinant proteins (Fig. 3C, Table 1).

**TABLE 1:** Immunodominant antigens of *B. duncani*

Organism	#	Antigen ID	Predicted function	GPI anchor	Residue	Molecular weight (kDa)
*B. duncani* Immunoreactive antigens	1	BdWA1_002112 (BdV4)	BdGPI12	Yes	356	52
	2	BdWA1_002869 (BdV19)	Unspecified product	No	–^*b*^	24.7
	3	BdWA1_003039 (BdV56)	Ubiquitin-like protein	No	–	20.6
	4	BdWA1_002223 (BdV234)	BdGPI13	Yes	219	27
	5	BdWA1_001369 (BdV86)	Unspecified product	No	–	31.7
	6	BdWA1_002154 (BdV23)	Unspecified product	No	–	30
	7	BdWA1_003373 (BdV235)	BdGPI17	Yes	103	12.5
	8	BdWA1_002224 (BdV2)	BdGPI14	Yes	496	58.8
	9	BdWA1_000266 BdWA1_000267^*a*^ (BdV1)	BdGPI1	Yes	475	57.4 56
	10	BdWA1_000750 BdWA1_003847^*a*^ (BdV83)	Unspecified product	No	–	75.9
	11	BdWA1_002873 (BdV35)	Unspecified product	No		30.1

^*a*^ Indicates member of a multigene family.

^*b*^ –, absence of a specific residue carrying the GPI anchor site on the protein.

### *B. duncani* immunodominant antigens are associated with parasite-derived vesicles

Of the 11 immunodominant antigens identified in this analysis, five were predicted to be GPI-anchored proteins including a previously reported antigen BdV234 (6). Among these candidates, BdV235 (BdWA1_003373) is a 127-amino acid protein predicted to contain a GPI anchor at residue 103. BdV235 is unique to *B. duncani* and lacks homologs in other *Babesia* species or related apicomplexan parasites. The *B. duncani* genome also encodes a closely related protein, BdWA1_003372, which shares ~70% sequence identity with BdV235, consistent with a potential gene duplication event.

**Fig 4:**
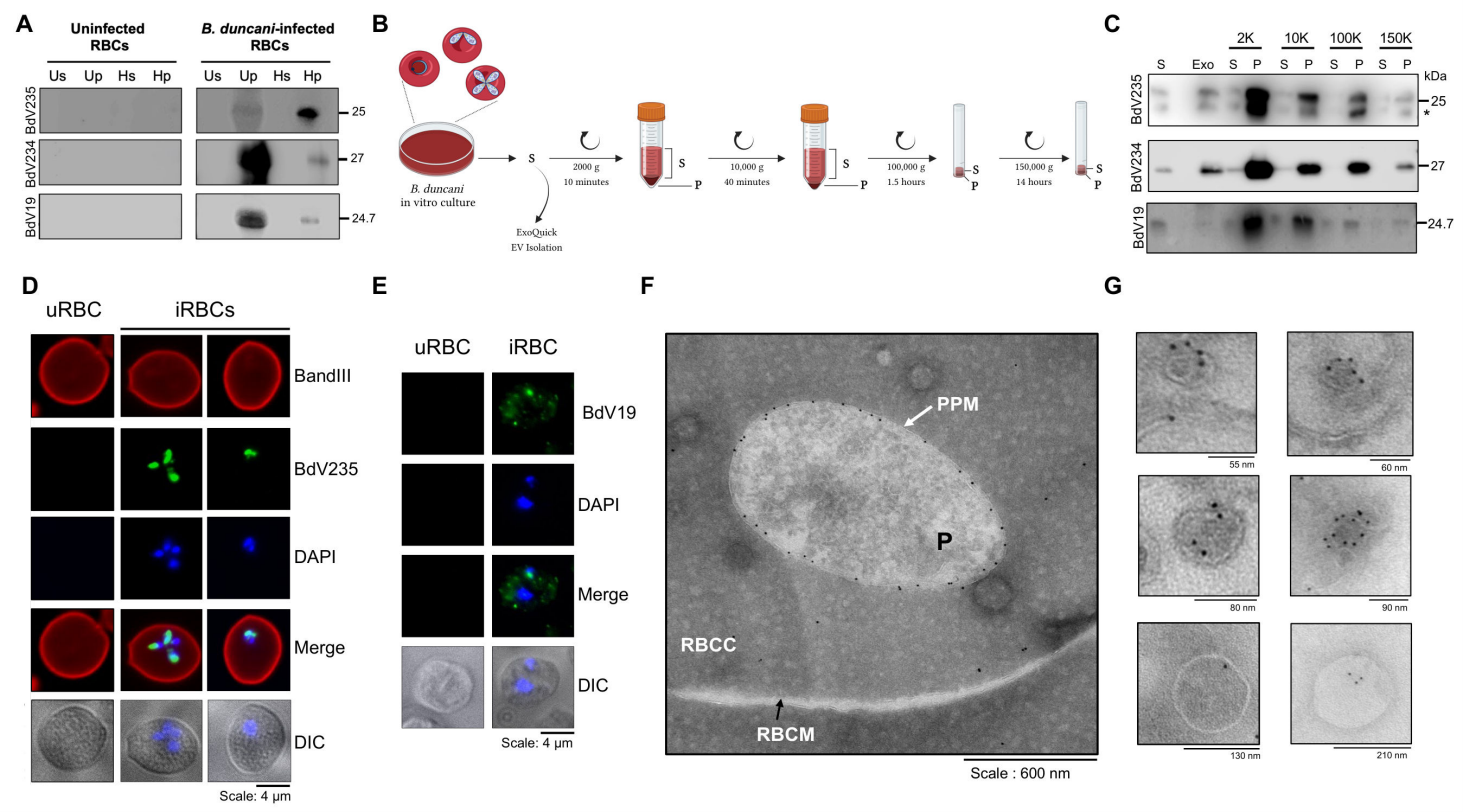
*B. duncani* immunodominant antigens associated with vesicular fractions. (**A**) Immunodetection of BdV235, BdV234, and BdV19 antigens in soluble and vesicle fractions from control uninfected or *B. duncani*-infected human RBCs. Us, culture supernatant; Up, vesicle-enriched ultracentrifuge pellet fraction from the culture supernatant; Hs, soluble cytosolic fraction from control uninfected or *B. duncani*-infected RBCs; Hp, vesicle-enriched ultracentrifuge pellet fraction from control uninfected or *B. duncani*-infected RBCs. (**B**) Schematic of vesicle fractionation: culture supernatant processed sequentially for exosome isolation and by centrifugation (2,000 × *g*, 10,000 × *g*, 100,000 × *g*, and 150,000 × *g*), yielding supernatant (S) and pellet (P) fractions. (**C**) Western blot analysis of fractions obtained by differential centrifugation probed with rabbit polyclonal antibody against BdV234, BdV235, and mouse sera (C3H/HeJ) against BdV19. * indicates a potentially processed form of BdV235. (**D**) Immunofluorescence staining of immunodominant antigen BdV235 using rabbit polyclonal antibody in control uninfected and *B. duncani-*infected human RBCs. BdV235 detected with goat anti-rabbit Alexa 488, RBC membranes with Band III, and parasite nuclei by DAPI. All experiments were conducted in biological triplicates. iRBCs, infected RBCs; uRBCs, uninfected RBCs. (**E**) Immunofluorescence staining of immunodominant antigen BdV19 using mouse sera (from *B. duncani-*infected C3H/HeJ mice) in control uninfected and *B. duncani-*infected human RBC. BdV19 detected with goat anti-mouse Alexa 488 and parasite nuclei by DAPI. All experiments were conducted in biological triplicates. iRBCs, infected RBCs; uRBCs, uninfected RBCs. (**F**) Immuno-electron microscopy of infected erythrocytes (high-pressure frozen, Durcupan-embedded) with anti-BdV235. (**G**) Immuno-EM of vesicles isolated from *B. duncani in vitro* culture, labeled with anti-BdV235. P, parasite; RBCC, red blood cell cytoplasm; RBCM, red blood cell membrane; DIC, differential interference contrast microscopy.

To examine the subcellular distribution of exported *B. duncani* antigens between soluble and vesicle-associated compartments, we analyzed three representative proteins: BdV235 and BdV19, two novel exported proteins unique to *B. duncani*, and BdV234, a previously characterized immunodominant antigen used as a positive control. Culture supernatants and host erythrocyte hemolysates were collected from uninfected and *B. duncani*-infected human erythrocyte cultures and subjected to ultracentrifugation at 120,000 × *g* for 14 h at 4°C. This approach generated four fractions: the culture supernatant-derived ultracentrifuge supernatant (Us), enriched in soluble components; the ultracentrifuge pellet (Up), containing vesicle-associated material; the hemolysate-derived ultracentrifuge supernatant (Hs), enriched in soluble cytosolic components; and the ultracentrifuge pellet (Hp), containing vesicle-associated material within the erythrocyte cytoplasm. Immunoblot analysis revealed that BdV235, BdV234, and BdV19 were detected exclusively in the vesicle-associated fractions (Up and Hp) derived from *B. duncani*-infected cultures and were absent from the corresponding soluble fractions (Us and Hs) as well as from all fractions obtained from uninfected controls (Fig. 4A).

To further define the association of these antigens with vesicles of different sizes, *B. duncani* culture supernatants were subjected to sequential differential centrifugation at 2,000 × *g*, 10,000 × *g*, 100,000 × *g*, and 150,000 × *g*, allowing enrichment of extracellular vesicles across a range of sizes (Fig. 4B). Supernatant and pellet fractions collected at each step were analyzed by immunoblotting using antibodies specific to BdV235, BdV234, and BdV19 (Fig. 4C). All three antigens were detected in total culture supernatants and in exosome-enriched fractions isolated using ExoQuick (Fig. 4C). Notably, each antigen consistently partitioned into pellet fractions at all centrifugation steps, indicating an association with vesicular material across multiple size classes (Fig. 4C). Collectively, these data demonstrate that BdV235, BdV234, and BdV19 are predominantly associated with *B. duncani*-derived vesicles (BDVs) rather than released as soluble antigens, both within infected erythrocytes and in the extracellular environment.

### Subcellular localization confirms vesicular export of immunodominant antigens

To further define the subcellular distribution of selected immunodominant antigens, immunofluorescence assays (IFA) were performed on *Babesia duncani*-infected human erythrocytes using preimmune sera and antibodies against BdV235 and BdV19, with uninfected erythrocytes serving as controls (Fig. 4D and E; Fig. S1). The data showed localization of BdV235 to the parasite as well as some foci observed in the cytosol of the infected erythrocyte, consistent with its association with BDVs (Fig. 4D). Similarly, BdV19 showed localization in both the parasite as well as host cytosol (Fig. 4E).

To achieve higher-resolution localization of BdV235, immunogold transmission electron microscopy was performed on *B. duncani*-infected erythrocytes. Gold labeling was predominantly observed at the parasite plasma membrane, with some signals detected in the surrounding erythrocyte cytosol (Fig. 4F). A quantitative analysis of BdV235 localization in the cytosol of *Babesia duncani*-infected versus uninfected erythrocytes reveals significantly greater localization in the infected cells. (Fig. S2). Furthermore, immuno-electron microscopy of vesicles isolated by ultracentrifugation revealed BdV235 labeling associated with both the lumen and membrane of parasite-derived vesicles ranging from 55 to 210 nm in diameter (Fig. 4G). Together, these findings indicate that BdV235 and BdV19 localize to parasite-associated membranes and vesicular structures and are present within vesicles released into the host erythrocyte cytoplasm.

## DISCUSSION

This study provides the first systematic evidence for a vesicle-mediated mechanism of antigen export in the human pathogen *B. duncani*, a virulent tick-borne apicomplexan parasite. Our results demonstrate that, during its asexual development both *in vitro* and in mice, *B. duncani* releases vesicles to traffic immunodominant antigens into the host erythrocyte cytosol and the infected cell environment. These findings unravel a unique mechanism by which *B. duncani* exports proteins to the host and identifies key antigens that may serve as diagnostic and therapeutic targets.

While C3H/HeJ and C57BL/6 mice show strikingly different survival outcomes after *B. duncani* infection, our work centers on mapping immunodominant antigens recognized by both strains rather than evaluating antibody titers as predictors of protection. These antigens are strongly recognized by IgG antibodies in both mouse models, though this humoral recognition does not necessarily translate to protective immunity. Previous studies demonstrate that C3H/HeJ mice succumb to infection due to proinflammatory cytokine responses rather than failure to mount antibodies (22, 23), whereas C57BL/6 mice can initially control parasitemia through both innate and adaptive mechanisms. Our serological profiling reveals key immunodominant antigens recognized during *B. duncani* infection and provides promising leads for diagnostics or vaccines, even as the link between immunodominance and disease severity or survival remains an open question in *B. duncani* pathogenesis.

The presence of vesicular structures in *B. duncani*-infected erythrocytes from *in vitro* cultures and infected animals suggests that vesicle-mediated export is a stable and intrinsic feature of *B. duncani* development. Similar vesicle-mediated export has been described in *B. microti* (18), whereas extracellular vesicles released from *B. divergens*-infected erythrocytes have also been characterized (19), suggesting that extracellular vesicle production may be a broader feature among *Babesia* species.

We systematically mined the *B. duncani* proteome to assemble a panel of recombinant proteins corresponding to known cell surface and secreted antigens expressed during the blood stage, focusing on extracellular proteins due to their direct accessibility to host immune surveillance and their potential as vaccine targets and serological diagnostic markers. Using this approach, we identified 11 unique immunodominant antigens and demonstrated that three of them (BdV235, BdV19, and BdV234) are exported by the parasite predominantly associated with BDVs. Using a combination of antigen screening, immunoblotting, immunofluorescence, and immuno-electron microscopy, we established that these proteins localize to the parasite plasma membrane and are subsequently associated with vesicles observed in the host cytoplasm. This mode of export contrasts sharply with the PEXEL-mediated protein trafficking in *P. falciparum* (24, 25), underscoring a divergent evolutionary strategy within the Apicomplexa for manipulating the host. The identification of vesicle-mediated export opens new avenues for the development of diagnostic and therapeutic tools.

The immunodominant antigens identified in this study are highly expressed during infection, positioning them as strong candidates for the development of advanced serological assays for *B. duncani*. Their vesicle association may enhance immunogenicity, making them attractive vaccine targets. Targeting vesicle formation or export machinery could disrupt parasite-host interactions, representing a novel therapeutic strategy. These findings raise important considerations for vaccine development. While vesicle-associated antigens may be naturally more immunogenic *in vivo* due to their mode of presentation, reproducing this with purified recombinant proteins may be challenging. Successful vaccine strategies may require delivery systems that mimic parasite-derived vesicle structures and immunologic features, such as nanoparticle encapsulation, liposomes, or engineered virus-like particles.

Our *B. duncani* antigen list (*n* = 105) included not only the canonical secreted proteins (those with classical signal sequences) and either identified as exported proteins by a Nanotrap-based proteomic approach (*n* = 10) or predicted to be surface-exposed antigens, but it also included typical cytosolic proteins (*n* = 25) that were exclusively identified by the Nanotrap-based methodology. A protein lacking classical secretion or membrane-targeting motifs, BdV19, was found to be immunodominant in this study. Immunoblot analyses confirmed the association of BdV19 with BDVs (Fig. 4C), suggesting that secretion of such immunodominant vesicle-associated cytosolic proteins may play an important role in *Babesia*-host interactions. Additionally, it is important to note that not all 105 *B. duncani* proteins were successfully expressed at detectable levels in our bacterial expression system. Some antigens are part of multigene families (indicated with star in Table 1) and hence were represented twice in our antigen library. Therefore, this study does not rule out the existence of additional immunodominant antigens in *B. duncani* that may have been missed due to expression limitations.

In summary, this study provides evidence that *B. duncani* uses a vesicle-mediated secretory mechanism to export immunodominant antigens into the host environment. This discovery elucidates a pivotal molecular mechanism underlying parasite-host interaction and advances the understanding of *Babesia* pathobiology. By identifying vesicle-associated antigens as immune targets, our findings strongly support their inclusion in future vaccine development efforts, highlighting them as prime candidates for inducing protective immune responses. Further research is needed to define BDV formation mechanisms, elucidate exported antigens’ roles in disease progression, and assess broader clinical and public health relevance.

## Data Availability

The data supporting the findings of the study are available within the article and supplemental material.

## SUPPLEMENTAL MATERIAL

Supplemental material iai.00763-25-s0001.docx Fig. S1 and S2.

ASM does not own the copyrights to Supplemental Material that may be linked to, or accessed through, an article. The authors have granted ASM a non-exclusive, world-wide license to publish the Supplemental Material files. Please contact the corresponding author directly for reuse.
