# Supplementary material for: Characterization of vesicle-associated exported immunodominant antigens of the human pathogen Babesia duncani
Source: Infect Immun. 2026 Jun 3;94(7):e00763-25. doi: 10.1128/iai.00763-25 (PMC13367061; doi:10.1128/iai.00763-25)
Supplement: Supplemental material — Fig. S1 and S2. [file iai.00763-25-s0001.docx]

**Supplementary material**

**Characterization of Vesicle-Associated Exported Immunodominant Antigens of the Human Pathogen *Babesia duncani***

Pallavi Singh^1^, Anasuya C Pal^1^, Jae-Yeon Choi^1^, Meenal Chand^1^, Pratap Vydyam^1^, Geeta Kumari^1^, Choukri Ben Mamoun^1 2 3 *^

^1^ Section of Infectious Diseases, Department of Internal Medicine, Yale School of Medicine, New Haven, Connecticut, , 06520, USA.

^2^ Department of Microbial Pathogenesis, Yale School of Medicine, New Haven, Connecticut, 06520 USA.

^3^ Department of Pathology, Yale School of Medicine, New Haven, Connecticut, 06520, USA.

^*^ Corresponding author: Choukri Ben Mamoun

Contact Address: Section of Infectious Diseases, Department of Internal Medicine, Yale School of Medicine, New Haven, Connecticut, 06520, USA

Email: [choukri.benmamoun@yale.edu](mailto:choukri.benmamoun@yale.edu)

**Figure S1. Immunofluorescence assay using BdV235 and BdV19 preimmune sera. A-B.** Immunofluorescence staining of uninfected and *B. duncani* infected human RBC using preimmune sera from rabbits used for raising polyclonal antibody against BdV235 (**A**) and preimmune sera from mice immunized with BdV19 (**B**). BdV235 preimmune sera was detected with goat anti-rabbit Alexa 488, and parasite nuclei by DAPI. BdV19 preimmune sera was detected with goat anti-mouse Alexa 488, and parasite nuclei by DAPI. PI, preimmune sera; DIC, Differential interference contrast microscopy.

**Figure S2. Quantification of BdV235 (immunogold particle density) in *Babesia duncani*-infected and uninfected erythrocytes.** Immunogold particle density in the cytosol of B. duncani-infected and uninfected red blood cells was measured as particles per square micrometer to assess BdV235 localization.
